# The Effect of Attentional Manipulation on Cough Reflex Sensitivity in Individuals with Refractory Chronic Cough and Healthy Controls

**DOI:** 10.3390/jcm14124199

**Published:** 2025-06-12

**Authors:** Jane R. Salois, Laurie J. Slovarp, Isabel Spinti, Jon Graham, Jethro Thorne, Amy Glaspey, Catherine Off, Marie Jetté

**Affiliations:** 1 School of Speech, Language, Hearing, & Occupational Sciences, University of Montana, 32 Campus Drive, Missoula, MT 59812, USA; jane.salois@umontana.edu (J.R.S.); catherine.off@umontana.edu (C.O.); 2 Department of Mathematical Sciences, University of Montana, 32 Campus Drive, Missoula, MT 59812, USA; jgraham@mso.umt.edu (J.G.);; 3 Department of Otolaryngology-Head and Neck Surgery, School of Medicine, University of Colorado Anschutz Medical Campus, 13001 East 17th Place, Aurora, CO 80045, USA

**Keywords:** cough reflex sensitivity, cough hypersensitivity syndrome, refractory chronic cough, capsaicin cough challenge testing

## Abstract

**Background:** Cough reflex sensitivity during cough challenge testing has been found to be modifiable with distraction in groups of healthy individuals. The purpose of this study was to examine this phenomenon in healthy controls and patients with refractory chronic cough (RCC) to advance our understanding of the role attention plays in cough modulation and shed light on avenues for therapeutic advances for RCC. **Methods**: Thirteen adults with RCC (mean age = 60, 12 women) and twelve healthy controls (mean age = 60, 11 women) participated in this study. The participants completed cough challenge testing with nebulized capsaicin doses tailored to their individual cough reflex sensitivity under distraction and no-distraction conditions. The distraction condition consisted of cough challenge testing while completing a cognitive (visual memory) task on a tablet. Capsaicin doses included the dose that elicited two coughs (C2), and up to three doubling doses above C2. Capsaicin doses were administered in serial order with a placebo dose randomized into the order to control for an anticipation effect during each condition. For each dose administered, the participants were instructed to cough if they needed to. Cough frequency within 15 s and maximal urge-to-cough with each dose were recorded. The order of conditions (distraction or no distraction) was alternated, and all testing was completed within one session. **Results**: There were no meaningful differences in the dose–response rate parameters for cough frequency or urge-to-cough, respectively, when comparing the results from the RCC group in the condition without distraction to the condition with distraction (*p* = 0.647, 95% CI = −2.25, 1.15; *p* = 0.783, 95% CI = −1.94, 0.84), and to the healthy control group without distraction (*p* = 0.921, 95% CI = −2.11, 2.73, *p* = 0.887, 95% CI = −1.40, 0.80), and with distraction (*p* = 0.970, 95% CI = −2.16, 3.36), *p* = 0.808, 95% CI = −1.49, 0.89). **Conclusions**: Distraction with the cognitive task chosen in this study did not influence cough reflex sensitivity in either group, which is contrary to studies on healthy volunteers and anecdotal evidence reported by RCC patients. Attentional resources may not have been sufficiently taxed, or too few capsaicin doses were administered to capture an effect as there was high individual variability in cough frequency and urge-to-cough. Additional research is needed to tailor the difficulty of the cognitive task to each participant and incorporate a real-world distraction scenario that may better reveal how attentional manipulation could be harnessed to optimize the effectiveness of behavioral cough suppression therapy for patients with refractory chronic cough.

## 1. Introduction

Refractory chronic cough (RCC), a cough lasting longer than eight weeks that has not responded to medical-guideline-based interventions, affects approximately seven million people in the United States every year [1,2]. RCC interferes with daily activities including answering the phone, eating, and sleeping [3], and is linked to high rates of depression [4,5], and urinary incontinence in women [6]. Cough hypersensitivity syndrome is accepted as a common underlying cause of RCC and is characterized by hypertussia (excessive coughing), allotussia (coughing in response to non-noxious and typically non-tussigenic stimuli), and laryngeal paresthesia (sensation of irritation in the throat area, also known as the urge-to-cough or UTC) [7,8,9,10,11].

While the exact mechanisms behind sensitization of the cough reflex are unknown, compelling brain imaging research highlights differences between patients with RCC and healthy controls and provides clues to understanding the phenomenon of increased cough reflex sensitivity in this population. Functional magnetic resonance imagining studies reveal anatomical and functional differences in the midbrain [12] and supra-medullary areas for UTC perception of nebulized capsaicin concentrations when UTC levels are matched to healthy controls [13]. In addition, there is evidence of impaired motor control of cough suppression networks in patients with RCC [13].

An examination of the neural networks involved in UTC perception and attention reveals significant overlaps. In Mazzone et al.’s (2007) work mapping neural networks involved in UTC perception, they identified activation of the anterior midcingulate cortex [14], which is highly correlated with sensory-driven urges such as the urge to void when the bladder is filled [15], the inferior and middle frontal gyri, and the insula [14] (believed to play a prominent role in visceral sensory processing or interoception [16]). Farrell et al. (2012) also described activation of the inferior parietal lobe and superior and middle frontal gyri as regions that could possibly reflect the attentional involvement and other higher order cognitive processes involved in the UTC network [17]. While more neuroimaging studies are needed to better elucidate these anatomic and functional neurologic differences in patients with RCC, an examination of existing behavioral research in healthy individuals complements this evidence that allocation of cognitive processing resources such as attention plays a role in influencing the cough reflex.

Behavioral research on voluntary control of the cough reflex suggests that the ability to intentionally modulate cough is present even when a very strong UTC is felt [18,19] and likely stems from an evolutionary adaptation to suppress cough in inappropriate situations and for survival [20]. Van den Bergh et al. (2012) reviewed how psychological factors impact cough regulation and laid the groundwork for considering the UTC sensation as an interoceptive process that may be impacted by cognitive factors such as attention [21]. A few observational studies exemplify how social context [22] and salience of airway irritation [23] modify cough control, and much attention is paid to the psychology of cough when considering the large placebo effect seen in cough research [24]. Recently, Slovarp et al. (2025) expanded on the intricacies of UTC interoception and the hypothesized role in RCC, calling for more research in this area to better understand how modifying UTC interoception via repeated cough suppression invokes neuroplasticity and changes cough reflex sensitivity [25].

Two groups of researchers have experimentally manipulated attention to examine the impact on cough reflex sensitivity in healthy controls and patients with Parkinson’s disease. Janssens et al. (2014) [26] hypothesized that an internal focus condition (i.e., increased attention to UTC interoception from counting their own coughs) would yield increased cough reflex sensitivity compared to an external focus condition where attention was divided between cough challenge testing with citric acid and a simultaneous auditory attention task (participants listened to high and low tones and were told to ignore high tones and count the low tones). As predicted, the participants coughed significantly more when counting their own coughs compared to in the divided attention condition (*p* = 0.001, η_p_^2^ = 0.37); however, in a repeated-measures analysis of variance, a main effect of attentional focus was seen in the first block of trials only (*p* = 0.035, η_p_^2^ = 0.18) [26]. A plausible rationale for this finding could be effects from short-term tachyphylaxis (temporary desensitization) following repeated doses of citric acid [27]. While the participants self-rated their UTC significantly higher during the interoceptive focus versus divided attention condition (*p* = 0.025, partial η_p_^2^ = 0.20), there was not a main effect of attentional focus on UTC (i.e., no conditional effect; *p* = 0.34) [26].

Perry and Troche (2019) [28] completed a similar study comparing cough reflex sensitivity with capsaicin cough challenge testing (concentrations of 0 μM, 10 μM, 25 μM, 50 μM, 100 μM, and 200 μM were presented in an independently randomized block order) with and without the same auditory attention task. They did not instruct participants to count their own coughs, as was carried out by Janssens et al. (2014), instructing them, instead, to cough if they needed to and then report their UTC [26]. The results closely followed the earlier findings of Janssens et al. (2014) [26]. The participants had significantly less coughing in the dual task condition with a conditional dual-tasking effect seen for the highest concentration of capsaicin using repeated measures-analysis of variance at the 200 μM dose (*p* = 0.001, η_p_^2^ = 0.78) [28]. Interestingly, when they examined a sample of patients with Parkinson’s disease using the same procedures, attentional manipulation with the same task did not result in changes to cough reflex sensitivity, beginning to reveal how allocation of cognitive processing resources and cough reflex sensitivity varies across clinical populations [29].

Investigating how attentional manipulation through distraction influences cough reflex sensitivity in patients with RCC has not been explored previously and is a key step toward understanding how attentional resources may regulate UTC. The allocation of attentional resources during dual-tasking is often explained by two prominent theoretical frameworks. The first, known as the capacity-sharing model, posits that cognitive processing resources are finite and distributed across tasks. As a result, when two tasks are performed simultaneously, the resources available for each are reduced, potentially impairing performance [30,31]. The second, the bottleneck model of attention, offers a related explanation, suggesting that tasks must pass through one or more cognitive bottlenecks, causing serial delays due to temporal limitations in processing. This model likewise accounts for impaired performance when task demands exceed attentional capacity [30].

The amount of attention directed toward interoceptive stimuli, such as the UTC, is theorized to be closely linked to an individual’s general tendency to notice, trust, and accurately interpret internal bodily sensations [31]. Moreover, attention to these internal cues likely influences the interoceptive feedback loop and associated behavioral responses [32]. Consequently, sustained attentional focus on UTC sensations in RCC may heighten or reinforce the maladaptive cough cycle (Janssens et al., 2014) [26]. Over time, repetition of this pattern may drive neuroplastic changes that further entrench cough hypersensitivity [25].

Patients with RCC often live with the persistent sensation of the UTC, which may result in a chronic pattern of heightened interoceptive awareness [25,26]. Clinically, many patients report reduced coughing during moments of deep engagement in tasks that demand their full attention. This observation aligns with the beneficial effects of distraction techniques commonly used in behavioral cough suppression therapy, supporting the hypothesis that diminishing attentional focus on interoceptive cues (by invoking the limits of cognitive processing resources via capacity sharing or bottlenecking) may reduce cough reflex sensitivity in individuals with RCC.

Investigating the impact of dual-tasking on cough reflex sensitivity could offer valuable insights for enhancing therapeutic approaches and refining behavioral treatment strategies. In this study, we aimed to assess the effects of attentional manipulation on cough reflex sensitivity in both patients with RCC and healthy controls. We hypothesized that a divided attention condition involving distraction would lead to reduced cough frequency and decreased UTC in both groups.

## 2. Methods

This study was submitted to the Food and Drug Administration (FDA) as a new study protocol amendment under Investigational New Drug (IND) #142148 and approved on 14 February 2022. University of Montana Institutional Review Board approval was granted on 29 March 2022 following full review by the committee under protocol #12-22. Data collection for the cohort of participants with RCC was conducted from April to July 2022 and data collection for the healthy cohort took place from September 2023 to February 2024.

Participants with RCC were recruited via phone and email contact with the first author. All participants with RCC were known to the first and second authors from participation in prior research in our lab. Healthy control individuals were recruited from a sample of convenience (individuals known to the first, second, or third authors) to match the gender and age distribution of the RCC group as closely as possible.

The participants first provided informed consent to participate in eligibility testing. After passing the eligibility testing criteria, they completed a separate informed consent for this experimental study. This two-step process was required as spirometry, part of the eligibility testing criteria, included some small risks to participants. The inclusion and exclusion criteria for eligibility testing and the experimental condition are presented in the Supplementary Materials (S1: Eligibility Criteria).

This study included two phases completed within one session (approximately ninety minutes total) with eligibility testing followed by the experimental procedures. In eligibility testing, spirometry was completed according to American Thoracic Society and European Respiratory Society guidelines [33] to determine normal lung function, defined as forced expiratory volume in one second (FEV1) divided by forced vital capacity (FVC) and FEV1 percent predicted of at least 0.7 [34], and three brief assessments were administered to ensure cognitive domains important to this study were in the average range (based on participant age) and to rule out a depressive disorder. Visual memory, mental flexibility, and depression severity were assessed as any baseline problems in these areas could potentially confound study results. The assessments included the Comprehensive Trail Making Test [35], the Wechsler Memory Scale-IV: Symbol Span [36], and the Patient Health Questionnaire-9 Item [37].

The experimental study involved four steps: (1) baseline testing with cough challenge testing (CCT) to determine the level of capsaicin that elicited two coughs (C2), (2) baseline testing with a visual working memory 2-back task, (3) CCT completed concurrently with the 2-back task (referred to as the “distraction” condition), and (4) CCT completed without any extraneous cognitive challenge (referred to as the “no distraction” condition). The participants completed the “distraction” and “no distraction” conditions in an alternating order based on enrollment in this study. Spirometry was repeated at the end of testing to ensure lung function remained normal [38].

### 2.1. Cough Challenge Testing (CCT) with Capsaicin

CCT is a valid and reliable means with which to test cough reflex sensitivity [39,40,41]. Capsaicin was chosen as the tussigenic agent as it is known to induce cough in a safe, dose-dependent, and reproducible manner [42]. CCT equipment was chosen based on European Respiratory Society guidelines to ensure a consistent and reproducible inspiratory dose with each breath [41]. This included a compressed-air-driven DeVilbliss 646 nebulizer with the straw and baffle assembly welded into place to optimize reproducibility [39], a QuarkSPIRO dosimeter (QuarkSPIRO, Cosmed, Pavona, Rome, Italy), and an inspiratory flow regulatory valve. Capsaicin used in this study was purchased from Formosa Laboratories, Inc. (Taoyuan, Taiwan) in accordance with FDA-IND specifications. The duration of aerosol delivery was 0.6 s, providing 0.002 mL concentration per inhalation. Capsaicin concentrations were made from diluted capsaicin stock (capsaicin diluted in ethanol to 0.001 M and 0.01 M concentrations) combined with 0.9% physiologic saline. All dilutions were prepared using sterile conditions under a laminar flow hood. All chemistry, manufacturing, and control (CMC) guidelines, outlined in FDA-IND #142148, related to the preparation of stock solutions and testing dilutions, and the storage of capsaicin were followed.

First, baseline CCT was completed to find C2, which was used to individualize the capsaicin concentrations administered in the CCT with and without 2-back conditions. The decision to tailor doses to each participants’ individual cough reflex sensitivity using their C2 level was influenced both by our lab’s prior work, where we observed significant variability in C2 levels for patients with RCC with a range of greater than seven doubling doses (0.49–31.25 uM) [43], and variability reported in the literature [39,44]. Given the significant variability, it was highly likely that preselection of four capsaicin concentrations for administration (as carried out by Perry & Troche 2019, 2021 [28,29]) would have been too difficult to tolerate for more sensitive individuals or not strong enough to elicit a response for others.

In the CTT procedures to find C2, aerosolized diluted capsaicin concentrations were delivered in serial doubling doses via a dosimeter-controlled nebulizer. The participants received physiologic saline as the first dose to prevent a startle effect, and then received 0.49 μM capsaicin concentration and the serial doubling doses above this until C2 was found. Each dose was separated by two minutes to prevent tachyphylaxis and two doses of physiologic saline were randomly interspersed as placebo trials to prevent a conditioned response in participants anticipating progressively higher doses [40,45]. The participants were instructed to hold the nebulizer in their non-dominant hand and to breathe normally (tidal breathing) in and out of the nebulizer mouthpiece. After three cycles of tidal breathing, the dose was administered. The participants were explicitly instructed to let their body react and to cough if needed, and to not suppress any coughs. Additionally, they were instructed to not talk for 15 s after inhalation as this may potentially suppress cough or result in more coughing in patients who are hypersensitive to vocalization [41]. They were carefully observed by the researcher and these directions were repeated if needed. The participants were instructed to take a drink of water after each trial. There was an approximately 20 min break between determining C2 and beginning CCT in the experimental procedures.

For the cough challenge testing (CCT) under both “no distraction” and “distraction” conditions, doses were administered under the same procedures described above. In each condition, the participants received their C2 dose followed by three subsequent doubling doses of capsaicin, presented in serial order. To reduce an anticipation effect, one randomized physiologic saline (placebo) dose was included among the five total doses. A random number generator determined in advance which dose would be the saline, and the participants were blinded to this assignment. The two conditions (“no distraction” and “distraction”) were administered in a counterbalanced order, with the sequence reversed for each successive participant. A five-minute rest period was provided between the conditions to minimize potential carryover effects.

In contrast to prior studies by Janssens et al. (2014) [26] and Perry and Troche (2019, 2021) [28,29], where participants received set concentrations of citric acid (30 mM, 100 mM, 300 mM, and 1000 mM) or capsaicin (10 µM, 25 µM, 50 µM, 100 µM, and 200 µM) in a random order, respectively, our methodology was designed to minimize the potential for short-term tachyphylaxis (desensitization)—a phenomenon which can occur when earlier administration of a higher dose blunts the response to subsequent lower doses [27,41,46]. This random dosing order in earlier studies may help explain why effects of attentional manipulation were only observed during the first block of administered concentrations. To address this, we administered the individualized capsaicin doses in ascending serial order alongside one randomized placebo dose.

We selected three doubling doses above each participant’s C2 threshold to minimize the likelihood of inducing acute tachyphylaxis [27]. This approach allowed all testing—including CCT for C2 determination and both experimental conditions (with and without distraction)—to be completed within a single session. Conducting testing in one session helped control for within-participant variability, particularly important for individuals with RCC, who often exhibit substantial day-to-day fluctuations in cough frequency, potentially reflecting changes in cough sensitivity [47].

Our decision was also informed by our lab’s prior observations that patients with RCC frequently experience increased discomfort at higher capsaicin concentrations [43]. We found it unlikely that most participants could tolerate more than three doubling doses above C2 across two administrations. To further address tolerability concerns, the participants rated the difficulty of each trial using a modified Borg scale, ranging from 0 (“no difficulty tolerating”) to 10 (“very, very difficult to tolerate”). If a participant rated any dose as 8/10 or higher, that dose and any higher concentrations were withheld.

### 2.2. Distraction Task: 2-Back

A visual working memory task completed on an iPad was chosen as a departure from the auditory attention tasks used in relevant prior studies [26,28,29]. This decision was made under the basic assumption that while inherently different constructs, by nature, any dual-tasking experience should tax attentional resources in much the same way across modalities [30,31]. A visual task was also preferable as we wanted to ensure the participants appropriately attended to the verbal instructions presented before each task without interference from anticipation of an upcoming auditory demand, and wanted a fully automated system for tracking performance with the cognitive task that would be easily replicable in future studies and may lend itself better to future adaptation of more salient, daily-life oriented tasks with a visual component (e.g., paying bills online or managing an online calendar).

N-back tasks are frequently used to operationalize working memory in neuroimaging studies [48,49]. Typically, the N-back task includes a stream of stimuli presented to an individual who then decides, for each stimulus item, if it matches the one presented “N” items ago (e.g., 1-back, 2-back, or 3-back). N-back is typically carried out with either visual or auditory stimuli and involves encoding of the stimuli, monitoring, maintaining, and updating of the material, and matching the current stimulus to the one that was presented “N” positions back. In general, as the levels of “N” increase, the number of errors increases [50]. There is also reliably increased activation in cortical areas commonly involved in working memory, including the prefrontal and parietal cortices [51,52]. N-back has construct validity for simple working memory as it has been shown to be highly correlated to a simple working memory task of digit span repetition [53,54].

A visual 2-back task was chosen to manipulate attention in this study. The 2-back level was selected over a 1-back or 3-back level as it was likely to elicit a challenge but would likely not be overwhelmingly difficult, where a flooring effect might be seen. The 2-back task was performed on an iPad using the Constant Therapy iOS application (www.constanttherapyhealth.com, access on 1 January 2020). On the app, this task is called “Remember pictures in order, N-back”. Constant Therapy is a validated cognitive–linguistic therapy platform [55]. The Constant Therapy version of this task delivers a randomly generated novel stream of colored pictures of various stimuli including common household and office items, animals, clothing, transportation, and food, with each picture separated by 2.28 s. The 2-back task was completed on an iPad. The participants completed five consecutive sets of the 2-back task. Each set contained 10 items for a total of 50 items. Task duration was approximately 2.25 min per set. Verbal directions were provided as follows, “I’ll show you a series of pictures. Tap when the picture is the same one you saw two pictures ago.” The directions were repeated as needed until the participant verbalized understanding of the task. Percent accuracy is calculated automatically within the app as the number of items completed correctly (selecting an incorrect picture or failing to select a correct picture are counted as errors) out of total number of items presented [56] and was recorded after completion of all five sets.

For the “distraction” condition where the 2-back task was completed concurrently with CCT, the participants were instructed to place the nebulizer mouthpiece in their mouth and simultaneously begin one set of the 2-back task on the iPad and tidal breathing. After eight seconds (approximately three cycles of tidal breathing), the dose was administered and the participant was reminded to let their body react naturally and cough if they needed, and to continue the 2-back task until the set was completed. The timing of 2-back set completion aligned almost exactly with the 15 s after dose administration. This coincided with the same window for which cough frequency and UTC data were recorded. These steps were repeated for all five doses. The percent accuracy on the 2-back task (the combined accuracy across the five sets) was recorded at the end of the procedures.

### 2.3. Outcome Measures

Data collection included cough frequency, UTC, and percent accuracy on the 2-back task in the distraction and no-distraction conditions. The number of coughs produced in the 15 s following capsaicin dose administration (coughs beyond this period may not be capsaicin-induced [19,40,57]) was manually counted by the researcher and recorded. This manual cough frequency count was verified by review of an audio/video recording in cases of possible ambiguity. The participant was then asked to rate their maximal UTC felt in association with the trial on a modified-Borg scale ranging from 0 “none” to 10 “very, very, very strong” [45]. The 2-back task accuracy was automatically calculated in the Constant Therapy application.

### 2.4. Statistical Analyses

R-4.4.2 (R Core Team, Vienna, Austria, 2021) and IBM SPSS Statistics (Version 29) were used for statistical analyses. Mean cough frequency and mean urge-to-cough rating as a function of capsaicin dose (1–4) were analyzed in a nonlinear logistic growth model to compare the dose–response curves for each condition between groups. This model was chosen as it provided a flexible way to quantify the relationship between the capsaicin dose (e.g., doses 1, 2, 3, 4, which were different concentrations as these were individualized to each participant) and the outcomes of cough frequency and urge-to-cough, particularly as this relationship was nonlinear. No corrections for multiple comparisons were made. Effect sizes were not included as this was a nonlinear model (wherein the effect of a predictor, such as dose, changes with the value of the predictor); thus, 95% confidence intervals for each model parameter were calculated. The deviance test was used to assess differences in dose–response parameters between groups. A Mann–Whitney U test was conducted to determine if there were differences in the 2-back task change scores between the RCC and healthy control groups.

## 3. Results

### 3.1. Participants

**Figure 1:**
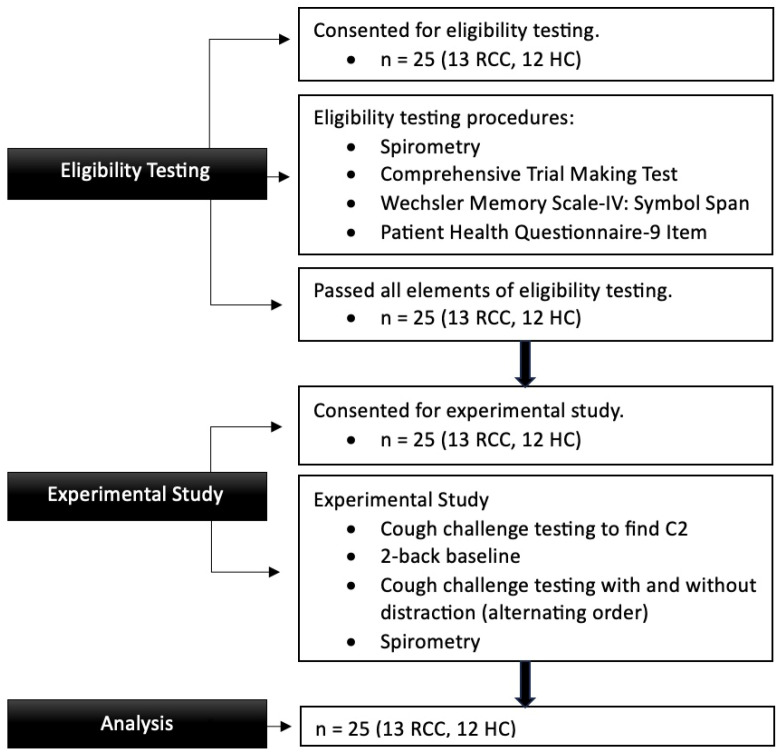
Participant Flow. Participant flow through all study phases. RCC = refractory chronic cough group, HC = healthy control group.

Thirteen adults with refractory chronic cough (mean age = 60, 12 women) and twelve healthy controls (mean age = 60, 11 women) participated in this study. Participant flow is shown in Figure 1.

Demographic characteristics, the capsaicin concentration that elicited at least two coughs (C2), and baseline and “distraction” conditions’ two-back task performance data are presented in Table 1.

### 3.2. Cough Frequency and UTC

There was no discrepancy between manual and recorded cough frequency counts for any trial (inter-rater reliability was 100%). There were several instances of missing data in the RCC group due to discomfort. If a participant rated their discomfort with any capsaicin dose an 8/10, indicating it was “difficult” to tolerate, they were not given that dose again or any higher doses. For the RCC group, participant RCC-2 was not administered capsaicin doses 3 or 4, participant RCC-3 was not given dose 4, and participant RCC-10 was not given doses 2–4. For the healthy control (HC) group, discomfort was less frequent and the only participant who did not complete all four doses was participant HC-1, who was not given dose 4. Descriptive statistics for each group and condition are presented in Table 2.

**Figure 2:**
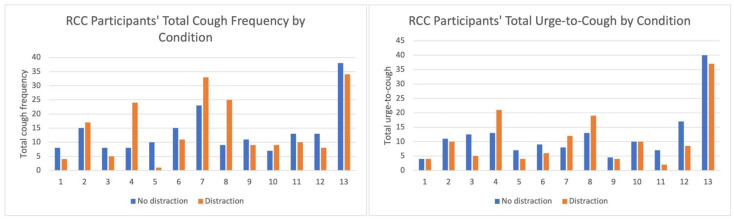
RCC participants’ total cough frequency and UTC. Total cough frequency and urge-to-cough (UTC) for the “no distraction” and “distraction” conditions for each refractory chronic cough (RCC) participant. The blue bar represents the “no distraction” condition where cough challenge testing was completed without an additional task, and the orange bars for the “distraction” condition represent the cough challenge testing plus two-back task condition results.

**Figure 3:**
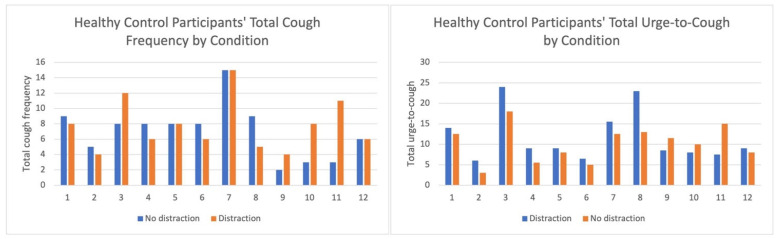
Healthy control participants’ total cough frequency and UTC. Total cough frequency and urge-to-cough (UTC) for the “no distraction” and “distraction” conditions for each healthy control participant. The blue bar represents the “no distraction” condition where cough challenge testing was completed without an additional task, and the orange bars for the “distraction” condition represent the cough challenge testing plus two-back task condition results.

Across all doses, the RCC group demonstrated higher mean cough frequency and UTC than healthy controls, with substantial inter-individual variability reflected by large standard deviations. Mean cough frequency and UTC generally increased with ascending capsaicin doses in both groups, with a few exceptions. RCC and healthy control group participants’ total cough frequency and UTC for each condition are presented in Figure 2 and Figure 3.

In the two-back task, the RCC group’s accuracy ranged from 55 to 100% in both conditions (mean = 78%, 79%, SD = 18, 17) for the baseline and “distraction” conditions, respectively. Accuracy for the healthy controls ranged from 17 to 100% (mean = 73%, SD = 28) at baseline, and from 53 to 97% (mean = 79%, SD = 14) in the “distraction” condition. The change in accuracy on the two-back task from baseline to the “distraction” condition was compared between the groups. For the RCC group, there were two outliers more than three standard deviations from the mean. The data were not normally distributed (assessed with Shapiro–Wilk’s test of normality, *p* = 0.02). For the healthy control group, there were no significant outliers and the data were normally distributed (test of normality was *p* = 0.093). Because of the data outliers and lack of normal distribution for the RCC group violating assumptions to conduct a *t*-test, a non-parametric test was selected. A Mann–Whitney U test was conducted to determine if there were differences in the two-back task change scores between the RCC and healthy controls. The distributions for the scores were similar, as assessed by visual inspection. No significant difference in median change scores was observed between groups (*U* = 83, *z* = 0.27, *p* = 0.81). Despite considerable variability, both groups maintained stable two-back task performance across conditions.

### 3.3. Dose–Response Modeling

Dose–response curves were used to compare mean cough frequency and UTC as a function of capsaicin dose (1, 2, 3, 4) and group (RCC or healthy control) for both the “no distraction” and “distraction” conditions. A logistic growth model was fit to all combinations of group and condition, and the model parameters were compared. In this model (shown below), the *E_m_* parameter was fixed at the mean response at capsaicin dose 4 to avoid overparameterizing the model. *E_m_* = 5.2, 5.1, 3.36, and 2.91 coughs for “RCC with no distraction”, “RCC with distraction”, “healthy controls with no distraction”, and “healthy controls with distraction”, respectively. With these designations, the model is expressed as follows:

$$E(y)=\frac{\left[E_{m}+\sum_{i=2}^{4}z_{i}*(\Delta E_{mi})]*[E_{0}+\sum_{i=2}^{4}z_{i}*(\Delta E_{0i})\right]}{\left[E_{0}+\sum_{i=2}^{4}z_{i}*(\Delta E_{0i})]+[(E_{m}-E_{0})+\sum_{i=2}^{4}z_{i}*(\Delta E_{mi}-\Delta E_{0i})]*\exp\{-(\delta +\sum_{i=2}^{4}z_{i}*\Delta \delta_{i})x\right\}}$$

where:
*y*=cough frequency/urge to cough.*x*=dose level (1, 2, 3, 4).$z_{i}$=0 if the case is in “RCC with no distraction”, 1 for case i where i=2,3,4 for “RCC with distraction”, “healthy controls with no distraction”, and “healthy controls with distraction”, respectively.*E*_*m*_=mean cough frequency at dose level 4 for the “RCC with no distraction” group.Δ*E*_*mi*_=change in cough frequency from the “RCC with no distraction” to group i=2,3,4 for “RCC with distraction”, “healthy controls with no distraction”, and “healthy controls with distraction”, respectively.E_0_=mean cough frequency at dose level 0 for the “RCC with no distraction” group.ΔE_0*i*_=mean cough frequency at dose level 0 for group i=2,3,4 for “RCC with distraction”, “healthy controls with no distraction”, and “healthy controls with distraction”, respectively.*δ*=dose–response rate parameter for “RCC with no distraction” condition.Δ$\delta_{i}$
=mean change in the dose–response rate parameter from the “RCC with no distraction” group to group i=2,3,4 for “RCC with distraction”, “healthy controls with no distraction”, and “healthy controls with distraction”, respectively.

**Figure 4:**
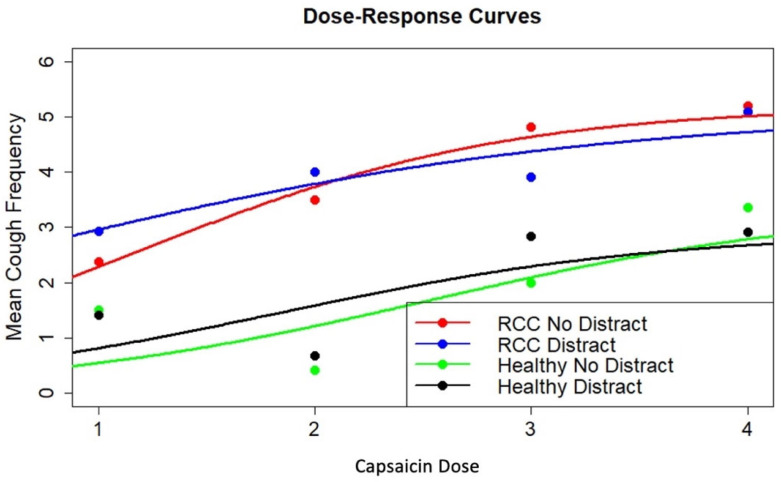
Dose–response curves for cough frequency. The dose–response curves for mean cough frequency by capsaicin dose (1, 2, 3, 4) for each group (refractory chronic cough (RCC) and healthy controls, referred to as “healthy”) and each condition (“distract” and “no distract”) are shown. “Distract” refers to the condition where cough challenge testing was paired with the two-back task, and “no distract” refers to the cough challenge testing condition without an additional task.

This is a nonlinear logistic growth model for four groups with eight parameters (all levels of the final four terms defined above) estimated using nonlinear least squares. This model was fit, producing the output in Table 3 and fitted dose–response curves through the mean responses shown in Figure 4. Running the model on 1000 bootstrap samples revealed asymmetric parameter estimate distributions. As a result, the bootstrap samples (generated using a stratified bootstrap) were used to generate bootstrap estimates of the parameters, and then the percentiles of these bootstrap distributions were used to estimate 95% confidence intervals for the eight model parameters.

Focusing parameters ΔE_02_, ΔE_03_ and ΔE_04_ in Table 3, there was no difference in the initial cough frequencies between the “RCC with no distraction” condition and the others, respectively (*p* = 0.550, *p* = 0.459, *p* = 0.560). There was also no evidence of a difference in the dose–response rate parameter δ between the “RCC with no distraction” condition and the others, respectively (*p* = 0.647, *p* = 0.921, *p* = 0.970). Bootstrap 95% confidence intervals contained zero for the final six parameters, further supporting the interpretation of no difference between conditions or groups. Within the RCC group, from Figure 4 and Table 3, the rate parameter δ was 0.44 units greater for the “no distraction” than the “distraction” condition, suggesting that there may have been a greater change in cough frequency across doses in the “no distraction” condition. But with only 10–13 individuals at each dose with high variability between individuals, this difference was not statistically significant. Using a deviance test on three degrees of freedom, there was no difference in the dose–response rate parameter between the four groups (*p* = 0.966) and no difference in the mean response at dose level 0 (E0) between the four groups (*p* = 0.575).

**Figure 5:**
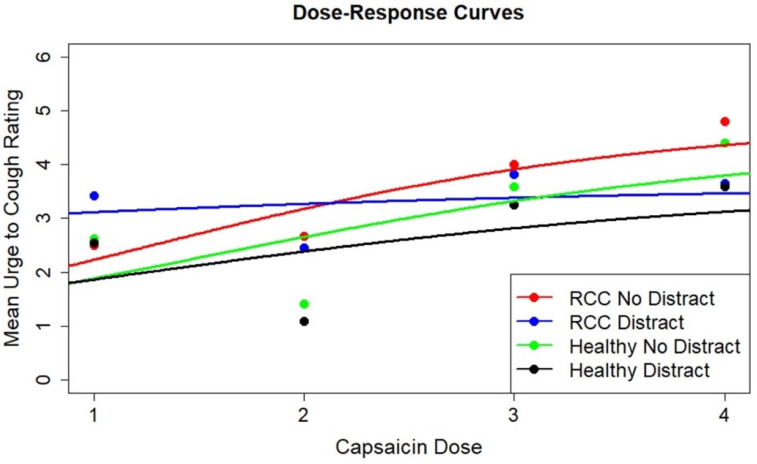
Dose–response curves for UTC. The dose–response curves for mean urge-to-cough (UTC) by capsaicin dose (1, 2, 3, 4) for each group (refractory chronic cough (RCC) and healthy controls, referred to as “healthy”) and each condition (“distract” and “no distract”) are shown. “Distract” refers to the condition where cough challenge testing was paired with the two-back task, and “no distract” refers to the cough challenge testing condition without an additional task.

To examine differences in UTC, a second nonlinear logistic growth model for the two groups and two conditions with eight parameters was estimated using nonlinear least squares. This model was fit, producing the output in Table 4 and fitted dose–response curves through the mean responses, as shown in Figure 5. Bootstrap confidence intervals were calculated using the same procedures already described.

Focusing on parameters ΔE_02_, ΔE_03_ and ΔE_04_ in Table 4, there was no difference in the initial mean UTC between the “RCC with no distraction” group with the others (“RCC with distraction”, “healthy controls with no distraction”, “healthy controls with distraction”), respectively (*p* = 0.415, *p* = 0.915, *p* = 0.996). There was also no evidence of a difference in the dose–response rate parameter δ between the “RCC with no distraction” condition and the others, respectively, (*p* = 0.783, *p* = 0.887, *p* = 0.808). Within the RCC group, from Figure 5 and Table 4, the rate parameter δ was 0.42 units greater for the “no distraction” than the “distraction” group, suggesting that there may have been a greater change in UTC across doses in the “no distraction” condition. But again, with only 10–13 individuals at each dose with high variability between individuals, this difference was not statistically significant. Using a deviance test on three degrees of freedom, there was no difference in the dose–response rate parameter between the four groups (*p* = 0.984) and no difference in the mean response at dose level 0 (E0) between the four groups (*p* = 0.881).

## 4. Discussion

Patients with RCC often report less frequent coughing while engrossed in a task and we have found distraction techniques to be complementary to cough suppression strategies in behavioral cough suppression therapy to help patients resist the UTC. Based on these observations, we hypothesized that a cognitive distraction task would reduce cough reflex sensitivity during capsaicin cough challenge testing. However, there was insufficient evidence of an effect in this study.

Although there was a modest trend toward increased coughing and increased UTC in the RCC group under the “no distraction” condition, consistent with our hypothesis, as evidenced by the increased growth rate parameter for the “RCC with no distraction” condition for both mean cough frequency and UTC, these differences were not statistically significant (*p* = 0.647 and *p* = 0.783). Similarly, performance on the two-back cognitive distraction task remained stable across conditions in both groups. Several methodological limitations likely contributed to these outcomes and should be considered when interpreting these results.

First, while we expected to find results for our healthy control group similar to those of Janssens et al.’s (2014) [26] and Perry & Troche’s (2019) [28] work with healthy controls, as we replicated several aspects of these study designs, there were key differences in the methodology and study procedures of these earlier works that may explain the lack of change in cough reflex sensitivity. Namely, the serial administration of up to four (depending on tolerability) capsaicin concentrations in each condition. While this likely helped reduce the risk of short-term tachyphylaxis, the serial dose administration with careful attention paid to participant discomfort also meant the highest doses were not administered to several individuals, limiting the total number of observations. Paired with an already small sample size, we may not have generated enough data points to demonstrate an effect between conditions.

In retrospect, it may have been a stronger design to complete CCT going beyond C2 to the highest level of capsaicin that could be tolerated by a participant and use this testing period as the control condition. This would have provided more data points and the ability to expand on the pharmacological dose–response curve analysis that was somewhat limited with only four doses to analyze. However, if this design was chosen, the order of conditions completed could not have been counter balanced as it was in this study, and the experimental phase of this study would have needed to be completed at least two hours after the control to reduce the risk of tachyphylaxis [27], creating a few more internal validity issues.

Second, a significant limitation of making a direct comparison of this work to the prior attentional manipulation studies is that the cognitive distraction chosen (two-back task) was a visual working memory task rather than an auditory attention task. In theory, this task should have taxed attentional resources in the same way since performance typically declines under multitasking conditions due to processing limitations [30]. However, had cognitive load been sufficiently taxed, differences in cough reflex sensitivity between conditions or performance on the two-back task, at least for the healthy control group, should have emerged.

This absence of a difference between conditions for the healthy control group as well as the group with RCC raises the question about appropriate challenge of the two-back task. By selecting the same difficulty level (two-back) for all participants, it is possible some individuals were more challenged with this than others. However, when examining both groups, there were only three participants who scored 100% on the task, which may have represented a ceiling effect, and otherwise, mean baseline accuracy for each group was lower than the 86% baseline accuracy reported on the auditory attention task by Perry & Troche (2019) [28]. The PI considered setting a minimal level of accuracy that would need to be met at baseline (e.g., 80%) to select the N-back level (e.g., 1-back or 2-back) but this was ultimately not included in this study’s procedures as this would have potentially introduced variability into the cognitive constructs recruited during the task, and potentially altered how cognitive processing resources were allocated in the two conditions. While this decision made it possible to examine the relationship between two-back task change across a wide range of accuracy and cough frequency, the small sample size limited the power of gaining a complete understanding of this relationship. Cognitive task selection should therefore be considered a study limitation as it is possible there would have been different results if all participants performed similarly at baseline on the two-back task (i.e., cognitive resources were taxed to the same degree).

Another consideration to generalizing results from this study is that the two-back task may not have been salient enough to the participant to capture the effect that attentional manipulation may have in daily life. The two-back task does not mimic activities carried out in daily living and it may be the case that emotional investment in a highly salient work or home-related task (e.g., cooking, working on an important project, reading, etc.) influences the allocation of cognitive resources differently. In functional magnetic resonance imaging studies examining UTC, the limbic system has an important and yet poorly understood role [14] and it may be that emotional stimulation relevant to the individual could result in a different effect on the sensorimotor processing of cough than was elicited with the two-back task used in this study. Additional research employing a functional task (e.g., cooking, reading) to examine the allocation of attentional resources toward the UTC is needed to further examine the impact patients living with RCC may encounter in their daily lives. Cough frequency monitoring with specific notation of activity or perception of engagement in the task would be a preliminary means to examine this in a real-world context.

It is also possible that the initial CCT used to determine the C2 dose produced a priming effect that heightened the salience of the two-back task during the distraction condition, thereby attenuating the effectiveness of the distraction. In other words, the participants may have anticipated experiencing an UTC simply due to the contextual cues associated with the procedure, and this expectation may have overridden the cognitive interference intended by the two-back task. This interpretation aligns with anecdotal reports from patients with RCC, who often note that they are more likely to cough when they are consciously thinking about or anxious about coughing, which would be unlikely when they are truly distracted in a real-world situation.

Any benefit of distraction may also be dependent on intensity—either on the intensity of the UTC or the intensity of the distraction stimulus. For example, it is possible that distraction is helpful up to a certain threshold of the UTC, after which the saliency of the UTC overrides any benefit of distraction. Alternatively, as seen with somatoform pain disorder, a stronger neural modulation effect in cortical pain processing regions is needed for somatoform pain patients to experience pain intensity reduction at a level similar to healthy controls in a dual-task paradigm [58]. If this same tenant applies to cough, the distraction would need to be highly engaging or salient to divert attentional resources away from the UTC.

Additional limitations to the generalizability of this study include the sampling methods used and the potential for inadvertent cough suppression by participants despite explicit instructions to refrain from suppressing and to allow coughs to occur naturally. We did not collect physiological data that could detect subtle breath-holding or covert suppression (e.g., respiratory monitoring), leaving open the possibility that some degree of cough suppression occurred. This is particularly plausible among participants in the RCC group, who had prior exposure to the concept of cough suppression through prior research participation. Both the RCC and healthy control groups were recruited via convenience sampling, which limits the external validity and rigor of the findings. With these considerations, we are tentative about drawing conclusions that distraction is not a means to modulate cough reflex sensitivity in patients with RCC. What remains unanswered by our study and warrants further investigation is what effect the intensity and saliency of a distraction stimulus may have on the UTC, and what effect may emerge with inclusion of a wider range of capsaicin concentrations to increase the sensitivity of a dose–response curve and subsequent analysis. Future research should also continue exploring the effect of intentionally directing an individual’s attention toward their UTC or away from it under distraction and no-distraction conditions (e.g., with use of physical or cognitive grounding techniques). This work could then expand to testing variable internal and externally focused attention in combination with cough suppression techniques (e.g., by increasing the intensity of UTC interoception or instead, directing a patient’s attention away from the UTC). Ultimately, research incorporating functional brain imaging studies with cough challenge testing with and without distraction is needed to better understand the allocation of cognitive resources and the impact on cough reflex sensitivity in this clinical population.

## 5. Conclusions

In this study, distraction using a two-back visual memory task did not significantly alter cough reflex sensitivity tested with capsaicin cough challenge in individuals with refractory chronic cough or healthy controls. Several limitations may have contributed to this outcome, including the limited number of capsaicin concentrations administered and the possibility that the selected distraction task did not sufficiently tax cognitive resources. Given that individuals with refractory chronic cough frequently report distraction as a helpful strategy for managing the urge-to-cough and a variety of cognitive techniques are frequently used in behavioral cough suppression therapy, further research is warranted to explore this phenomenon. A deeper understanding of how attentional resource allocation influences the interoceptive mechanisms involved in RCC may inform future refinement of behavioral cough suppression therapy.

## Figures and Tables

**Table 1:** Participant demographics, C2, and two-back results.

Participant ID	Age	Sex	C2	Baseline Two-Back % Accuracy	Two-Back % Accuracy During CCT
RCC-1	75	F	0.49 µM	61%	69%
RCC-2	73	F	15.63 µM	78%	69%
RCC-3	57	F	31.25 µM	60%	85%
RCC-4	65	F	3.91 µM	55%	54%
RCC-5	54	M	1.95 µM	95%	94%
RCC-6	72	F	1.95 µM	73%	73%
RCC-7	67	F	7.81 µM	60%	56%
RCC-8	44	F	0.98 µM	100%	89%
RCC-9	58	F	0.49 µM	100%	96%
RCC-10	44	F	3.91 µM	100%	100%
RCC-11	67	F	0.49 µM	78%	97%
RCC-12	52	F	7.81 µM	97%	92%
RCC-13	63	F	15.63 µM	56%	55%
Group mean (SD):	60.9 (10.4)			78% (18.4%)	79% (17.2%)
HC-1	56	F	0.98 µM	100%	97%
HC-2	77	F	31.25 µM	56%	53%
HC-3	81	M	3.91 µM	84%	64%
HC-4	59	F	3.91 µM	29%	77%
HC-5	49	F	0.49 µM	74%	75%
HC-6	53	F	7.81 µM	92%	93%
HC-7	62	F	1.95 µM	88%	76%
HC-8	57	F	7.81 µM	17%	71%
HC-9	58	F	0.98 µM	100%	75%
HC-10	42	F	3.91 µM	53%	69%
HC-11	70	F	0.98 µM	81%	95%
HC-12	62	F	1.95 µM	100%	97%
Group mean (SD):	60.5 (11.1)			73% (28.2%)	79% (14.2%)

RCC = refractory chronic cough group, HC = healthy control group, C2 = concentration of capsaicin that elicited at least two coughs.

**Table 2:** Group mean cough frequency and UTC results by condition and dose.

	Dose	UTC Mean (SD)		Cough Frequency Mean (SD)	
		No Distraction	Distraction	No Distraction	Distraction
RCC	1	2.46 (3.77)	2.65 (3.27)	2.38 (3.31)	3.69 (4.57)
	2	2.67 (2.80)	2.46 (2.97)	3.50 (3.45)	4.00 (4.73)
	3	4.00 (3.61)	4.08 (3.60)	4.82 (5.17)	3.91 (3.45)
	4	4.80 (2.94)	3.65 (2.89)	5.20 (3.61)	5.10 (4.20)
Healthy control	1	2.63 (1.65)	2.54 (2.18)	1.50 (1.31)	1.42 (1.44)
	2	1.42 (1.81)	1.08 (1.46)	0.42 (0.90)	0.67 (1.23)
	3	3.58 (3.08)	3.25 (2.80)	2.00 (2.09)	2.83 (2.76)
	4	4.41 (2.73)	3.59 (1.39)	3.36 (2.01)	2.91 (2.07)

RCC = refractory chronic cough group, Dose = capsaicin dose, SD = standard deviation.

**Table 3:** Coefficients for cough frequency dose-response analysis.

Parameter	Estimate	SE	*t*-Value	*p*-Value	95% (Bootstrap) Confidence Intervals
E_0_	1.01	1.00	1.009	0.314	(0.04, 3.62)
δ	1.18	0.74	1.594	0.113	(0.13, 2.86)
ΔE_02_	1.02	1.70	0.600	0.550	(−0.81, 3.41)
Δδ_2_	−0.44	0.96	−0.459	0.647	(−2.25, 1.15)
ΔE_03_	−0.80	1.08	−0.742	0.459	(−3.25, 0.66)
Δδ_3_	−0.11	1.08	−0.100	0.921	(−2.11, 2.73)
ΔE_04_	−0.69	1.18	−0.584	0.560	(−3.14, 0.94)
Δδ_4_	−0.05	1.25	−0.038	0.970	(−2.16, 3.36)

**Table 4:** Coefficients for UTC dose–response analysis.

Parameter					Estimate
E_0_					1.34
δ	0.81	SE	*t*-value	*p*-value	95% (bootstrap) confidence intervals
ΔE_02_	1.58	1.03	1.293	0.198	(0.08, 3.52)
Δδ_2_	−0.42	0.55	1.480	0.141	(0.06, 2.00)
ΔE_03_	−0.15	1.93	0.817	0.415	(−1.00, 3.11)
Δδ_3_	−0.11	1.53	−0.276	0.783	(−1.94, 0.84)
ΔE_04_	−0.01	1.41	−0.107	0.915	(−2.23, 1.66)
Δδ_4_	−0.20	0.74	−0.143	0.887	(−1.40, 0.80)

SE = standard error.

## Data Availability

The data presented in this study are available on request from the corresponding author.

## Supplementary Materials

The following supporting information can be downloaded at: https://www.mdpi.com/article/10.3390/jcm14124199/s1, S1: Eligibility criteria [59,60].
