# Supplementary material for: The Effect of Attentional Manipulation on Cough Reflex Sensitivity in Individuals with Refractory Chronic Cough and Healthy Controls
Source: J Clin Med. 2025 Jun 12;14(12):4199. doi: 10.3390/jcm14124199 (PMC12194027; doi:10.3390/jcm14124199)
Supplement: Supplementary file 1 [file jcm-14-04199-s001.zip › jcm-3618874-supplementary.pdf]

Supplemental Materials

S1: Eligibility Criteria

Eligibility Testing

Inclusion Criteria:

- At least 18 years old.
- Suffering from a cough lasting at least eight weeks.
- Self-report of receiving the following assessments for current cough symptoms with unremarkable results:
  - Physical evaluation by at least one physician.
  - Chest x-ray.
  - Laryngoscopic examination by a qualified otolaryngologist or speech-language pathologist.

Exclusion Criteria:

- Current smoker of any substance.
- Diagnosis of any of the following:
  - Respiratory disease (e.g., COPD, asthma)
  - Neurobehavioral disorder (e.g., Attention-Deficit-Hyperactivity Disorder)
  - Neurogenic disease (e.g., Parkinson's disease, cerebrovascular disease)
  - Head and neck cancer
- Use of the following medication within the specified amount of time:
  - Angiotensin-converting enzyme inhibitor (ACE-I) in the past four weeks.
    - ACE-Is include: Benzapril (Lotensin), Captopril (Capoten), Enalapril/Enalaprilat (Vasotec oral and injectable), Fosinopril (Monopril), Lisinopril (Zestril and Prinivil), Moexipril (Univasc), Perindopril (Aceon), Quinapril (Accupril), Ramipril (Altace), and Trandolapril (Mavik).
  - Neuromodulator medication in the past 48 hours.
    - Neuromodulators include medications such as Neurontin (Gabapentin) and amitriptyline.
- Tested positive for COVID-19 (or presented with COVID-19 symptoms without testing) within the past 10 days. (Participants who had symptoms within 10 days and tested negative for COVID-19, were allowed to enroll if they passed the following COVID-19 screening questions.)
- Answer "yes" to any of the following COVID-19 screening questions:
  - Have you had any of the following symptoms of COVID-19 in the past 48 hours?
    - Fever or chills, sputum, difficulty breathing, fatigue, muscle or body aches, headache, loss of taste or smell, sore throat, congestion or runny nose, nausea or vomiting, diarrhea.
    - Is your cough any different than it was prior to the COVID-19 outbreak (December 2019)?
    - Have you been told by a public health official to isolate due to COVID-19 exposure?
    - Have you been in contact with any person known to have COVID-19 or

with active COVID-19 symptoms (as described above) within the past 10 days?
    - Fever of greater than 100.4□F.
    - Have not followed current local, state, and CDC COVID-19 mitigation guidance.
    - Unwilling to comply with COVID-19 precautions.

Experimental Testing

Inclusion Criteria

- Must have passed all components of the eligibility testing study with normal results.
    - Spirometry with normal lung function for age, height, weight, and sex.
        - Normal lung function is defined as FEV1/FVC ration and FEV1 percent predicted of at least 0.7.
    - Neuropsychological assessments completed with scores falling within the normal range for age and educational level (as appropriate).
        - Wechsler Memory Scales-IV: Symbol Span
        - Comprehensive Trail Making Test
        - Patient Health Questionnaire- 9-Itme
    - At least 18 years old.

Exclusion Criteria

- Current smoker of any substance.
- Pregnant (willing to take pregnancy test if needed to confirm).
- Diagnosis of any of the following:
    - Respiratory disease (e.g., COPD, asthma)
    - Neurobehavioral disorder (e.g., Attention-Deficit-Hyperactivity Disorder)
    - Neurogenic disease (e.g., Parkinson's disease, cerebrovascular disease)
    - Head and neck cancer
- Use of the following medication within the specified amount of time:
    - Angiotensin-converting enzyme inhibitor (ACE-I) in the past four weeks.
        - ACE-Is include: Benzapril (Lotensin), Captopril (Capoten), Enalapril/Enalaprilat (Vasotec oral and injectable), Fosinopril (Monopril), Lisinopril (Zestril and Prinivil), Moexipril (Univasc), Perindopril (Aceon), Quinapril (Accupril), Ramipril (Altace), and Trandolapril (Mavik).
    - Neuromodulator medication in the past 48 hours.
        - Neuromodulators include medications such as Neurontin (Gabapentin) and amitriptyline.
- Tested positive for COVID-19 (or presented with COVID-19 symptoms without testing) within the past 10 days. (Participants who had symptoms within 10 days and tested negative for COVID-19, were allowed to enroll if they passed the following COVID-19 screening questions.)
- Answer "yes" to any of the following COVID-19 screening questions:
    - Have you had any of the following symptoms of COVID-19 in the past 48 hours?
        - Fever or chills, sputum, difficulty breathing, fatigue, muscle or body aches, headache, loss of taste or smell, sore throat, congestion or runny nose, nausea or vomiting, diarrhea.
        - Is your cough any different than it was prior to the COVID-19 outbreak

(December 2019)?

- Have you been told by a public health official to isolate due to COVID-19 exposure?
- Have you been in contact with any person known to have COVID-19 or with active COVID-19 symptoms (as described above) within the past 10 days?
- Fever of greater than 100.4□F.
- Have not followed current local, state, and CDC COVID-19 mitigation guidance.
- Unwilling to comply with COVID-19 precautions.
